# MEG correlates of temporal regularity relevant to pitch perception in human auditory cortex

**DOI:** 10.1016/j.neuroimage.2022.118879

**Published:** 2022-04-01

**Authors:** Seung-Goo Kim, Tobias Overath, William Sedley, Sukhbinder Kumar, Sundeep Teki, Yukiko Kikuchi, Roy Patterson, Timothy D. Griffiths

**Affiliations:** a Department of Psychology and Neuroscience, Duke University, Durham, NC 27708, USA; b Duke Institute for Brain Sciences, Duke University, Durham, NC 27708, USA; c Center for Cognitive Neuroscience, Duke University, Durham, NC 27708, USA; d UCL Ear Institute, University College London, London WC1X8EE, UK; e Wellcome Trust Centre for Human Neuroimaging, Institute of Neurology, University College London, London WC1N 3BG, UK; f Biosciences Institute, Faculty of Medical Sciences, Newcastle University, Newcastle upon Tyne NE2 4HH, UK; g Department of Physiology, Development and Neuroscience, University of Cambridge, Cambridge CB2 3EG, UK; h Research Group for Neurocognition of Music and Language, Max Planck Institute for Empirical Aesthetics, Grueneburgweg 14, Frankfurt/Main 60322, Germany; i Human Brain Research Lab, Department of Neurosurgery, University of Iowa, Iowa City , Iowa 52242, USA

**Keywords:** Pitch, Regularity, Auditory cortex, Heschl's gyrus, Planum temporale, MEG

## Abstract

We recorded neural responses in human participants to three types of pitch-evoking regular stimuli at rates below and above the lower limit of pitch using magnetoencephalography (MEG). These bandpass filtered (1–4 kHz) stimuli were harmonic complex tones (HC), click trains (CT), and regular interval noise (RIN). Trials consisted of noise-regular-noise (NRN) or regular-noise-regular (RNR) segments in which the repetition rate (or fundamental frequency F0) was either above (250 Hz) or below (20 Hz) the lower limit of pitch. Neural activation was estimated and compared at the senor and source levels.

The pitch-relevant regular stimuli (F0 = 250 Hz) were all associated with marked evoked responses at around 140 ms after noise-to-regular transitions at both sensor and source levels. In particular, greater evoked responses to pitch-relevant stimuli than pitch-irrelevant stimuli (F0 = 20 Hz) were localized along the Heschl's sulcus around 140 ms. The regularity-onset responses for RIN were much weaker than for the other types of regular stimuli (HC, CT). This effect was localized over planum temporale, planum polare, and lateral Heschl's gyrus. Importantly, the effect of pitch did not interact with the stimulus type. That is, we did not find evidence to support different responses for different types of regular stimuli from the spatiotemporal cluster of the pitch effect (∼140 ms).

The current data demonstrate cortical sensitivity to temporal regularity relevant to pitch that is consistently present across different pitch-relevant stimuli in the Heschl's sulcus between Heschl's gyrus and planum temporale, both of which have been identified as a “pitch center” based on different modalities.

## 1 Introduction

Pitch is a ubiquitous property of many natural sounds (Plack and Oxenham, 2005) and an important cue in human communication signals, such as speech and music. In vocalizations such as vowels, the vibrations of the vocal cord produce regular sound waveforms that are associated with a certain pitch. In music, sequencing pitches gives rise to melodies, while the particular relationships between pitches form the foundation of Western tonal harmony.

Behaviorally, it has been shown that a temporally regular sound with a repetition rate, or fundamental frequency (F0), greater than 30 Hz is perceived as having pitch (de Cheveigné, 2006; Krumbholz et al., 2003; Pressnitzer et al., 2001). For example, when the repetition rate of a click train is above 30 Hz, a pitch is perceived that corresponds to the repetition rate. In contrast, a click train with a repetition rate below 30 Hz is perceived as individual, rapidly repeating clicks, but it does not evoke a pitch percept. Thus, 30 Hz is commonly denoted as the lower limit for pitch perception. This holds for regular waveforms with very different acoustic characteristics, e.g., harmonic complex tones with different phase, click trains, or regular-interval noise (Krumbholz et al., 2003; Pressnitzer et al., 2001). Note that spectral energy at F0 is not necessary to evoke a pitch of F0 (e.g., “missing fundamental”; see Licklider (1954)).

The neural basis of pitch in humans is still debated, and largely centers on the question whether there exists a specific cortical area with dedicated neurons that gives rise to a pitch percept (a ‘pitch center’ or ‘pitch extractor’), or whether the percept arises through a distributed system over multiple cortical areas (Griffiths and Hall, 2012; Kumar et al., 2011). It has been suggested that a “pitch extractor” should be (1) sensitive to the existence of an F0 and changes in F0 that are relevant to pitch (i.e., above 30 Hz in humans), and (2) invariantly so across other stimulus dimensions (de Cheveigné, 2006). Previous electrophysiological studies found neural correlates that satisfy the first criterion in primary and non-primary auditory areas in humans and non-human primates using a few types of regular stimuli. Bendor and Wang (2005) demonstrated that single neurons in anterolateral A1 and adjacent belt regions of marmoset monkeys show similar responses to sine waves and missing fundamental harmonic complex tones with the same F0, from which different subsets of neurons were sensitive to different types of regular stimuli such as click-trains and RIN. In relation to this and human neuroimaging studies, the lateral Heschl's gyrus (HG) in human auditory cortex, a non-primary auditory region anterolateral to primary auditory cortex, has been suggested as one of the ‘pitch center’ candidates (Bendor and Wang, 2006).

Non-invasive neuroimaging studies in humans have found pitch-selectivity in belt- and parabelt-equivalent regions, including lateral HG, lateral planum temporale (PT) and lateral superior temporal gyrus (STG) (Griffiths, 2005; Patterson et al., 2002; Penagos et al., 2004). Hall and Plack (2009) reported blood-oxygenation-level dependent (BOLD) activation in the lateral HG and PT across various pitch-inducing stimulus types, including harmonic complex tones, Huggins tones, and iterative rippled noise (IRN; Yost (1996)), or regular-interval noise (RIN). However, their data showed that RIN-related activation in lateral HG was potentially due to slow spectrotemporal modulations inherent in the RIN stimulus (Barker et al., 2012), suggesting that the activation in the lateral HG may not be specific to pitch perception. A preference for resolved harmonics in more anterolateral auditory areas such as the planum polare (PP) and lateral STG has also been reported (Norman-Haignere et al., 2013).

Intracortical depth recordings from a single human patient revealed that an electrode placed around the tip of lateral HG showed strong pitch-onset responses in the absence of noise-onset responses, while an electrode in the medial portion of HG showed the opposite pattern – i.e., the absence of pitch-onset responses and strong noise-onset responses (Schönwiesner and Zatorre, 2008). Griffiths et al. (2010) reported evoked responses and gamma oscillations for pitch-relevant regular stimuli in human auditory cortex, particularly from electrodes located in HG, maximally in the medial region, a primary core area, but also including the lateral region. Furthermore, two recent electrophysiological studies with wider coverage provide converging evidence for a broader, distributed representation of pitch processing in auditory cortex. Kikuchi and colleagues reported similar evoked and induced local field potential (LFP) responses as well as single-unit responses to two different types of broadband pitch (harmonic complex tones and RIN) in core and belt auditory regions of the rhesus macaque (Kikuchi et al., 2019). Similarly, Gander and colleagues found comparable intracranial LFP responses localized in the core and belt equivalents of eight human patients, supporting the idea of distributed neural ensembles for pitch-related processing (Gander et al., 2019).

The overall diversity of findings may arise from the diversity in species (e.g., humans, marmosets, macaques, ferrets), sources of neural signals (e.g., invasive and non-invasive electrophysiology, hemodynamics), and particular stimulus properties (e.g., harmonic complex tone, click train, RIN, missing fundamental pitch). In the current study, we took advantage of the variety in stimulus properties and used three different pitch-evoking stimulus types (harmonic complex, HC; click train, CT; regular-interval noise, RIN) to determine a pitch response that is invariant across pitch types. In addition, in order to separate pitch responses from more general responses to periodicity or regularity, we employed repetition rates either above (250 Hz) or below (20 Hz) the lower limit of pitch. Finally, we concatenated noise and regular stimulus segments to separate pitch responses from low-level sound-onset responses: trials consisted of noise-regular-noise (NRN) or regular-noise-regular (RNR) segments in which the repetition rate (or fundamental frequency F0) in the regular segments was either above (250 Hz) or below (20 Hz) the lower limit of pitch. This paradigm enabled us to investigate a main effect of pitch (250 Hz vs. 20 Hz), its invariance to different pitch types (HC, CT, RIN), as well as potential interactions. Recording magnetoencephalography (MEG) in human participants, we show source-localized evoked responses to pitch-evoking stimuli with a characteristic latency of ∼140 ms in areas distributed over auditory cortex.

## 2 Materials and methods

### 2.1 Participants

Twenty right-handed participants took part in the study. All participants provided written consent in accordance with the local research ethics committee at University College London and reported normal hearing with no neurological history. One participant was excluded for poor dipole fitting for head position coils (goodness of fit < 0.85), thus 19 participants (mean age = 25.9 ± 5.5, 8 females) were included in sensor-level analyses. From thirteen participants with structural magnetic resonance imaging (MRI) data, three participants were excluded for problems in head reconstruction (i.e., skin isosurfaces), which did not allow manual coregistration between MEG and MRI data, leaving 10 participants (mean age = 26.2 ± 6.5, 5 females) that were included in source-level analyses.

### 2.2 Stimuli and paradigm

All stimuli were created in the digital domain using MATLAB (RRID:SCR_001622^1^) with a sampling rate of 44.1 kHz and 16-bit resolution.

We used three types of pitch-evoking regularity: (1) harmonic complex tones (HC) with harmonic series up to the Nyquist frequency in positive Schroeder phase; (2) click trains (CT) with a click duration of 1 sample (1/44,100 s); and 3) regular interval noise (RIN) using the add-new procedure with a gain of 1 and 16 iterations (Yost, 1996). The fundamental frequency was either F0 = 250 Hz or F0 = 20 Hz, i.e., either well above or below the lower limit of pitch (Pressnitzer et al., 2001). Samples in the noise segments were taken from a zero-mean unit-variance Gaussian distribution (*randn* in MATLAB).

**Fig. 1:**
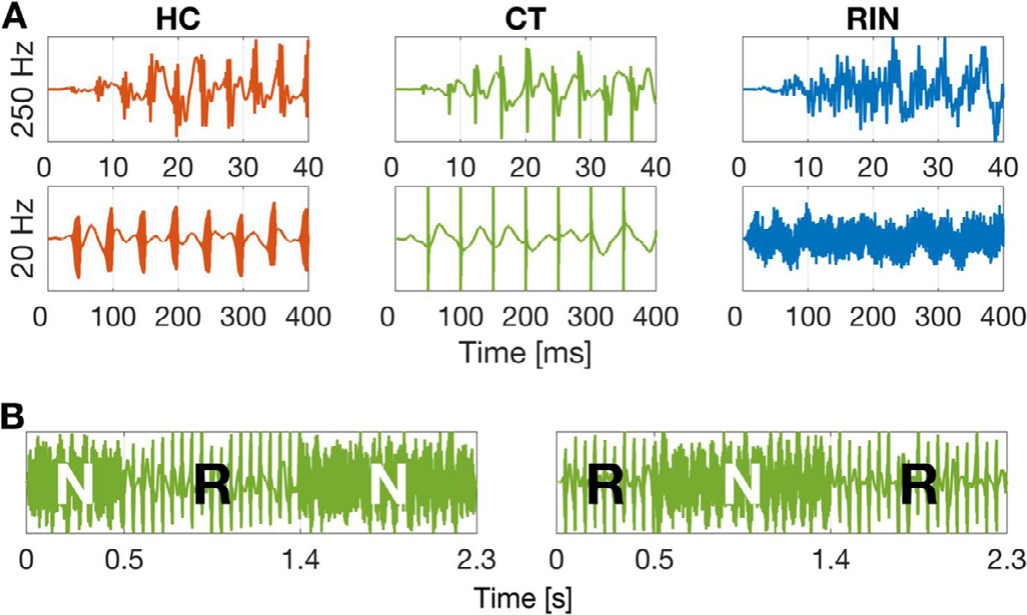
*Stimulus examples.* (A) Waveforms of regular stimuli. HC, Harmonic complex; CT, click-train; RIN, regular-interval noise. (B) Waveforms of Noise-Regular-Noise (NRN) or Regular-Noise-Regular (RNR) sequences of CT-20 Hz.

Each stimulus consisted of three contiguous segments of 500 ms, 900 ms, and 900 ms in duration (2300 ms trial length). Stimuli were constructed by concatenating the three segments in two ways: noise-regular-noise [NRN], or regular-noise-regular [RNR]. The stimuli were bandpass filtered between 1 and 4 kHz using a 4th order Butterworth filter. A masking noise with a bandwidth of 0.5*F0 to 1.5*F0 Hz using a 2nd order Butterworth filter was created to mask potential distortion products around F0 and then added to the signal. Finally, the entire NRN or RNR sequences were filtered to impose a 1/f pink power spectrum using filter coefficients^2^ and then windowed with raised cosine on and off ramps (20 ms; 0–0.020 s and 2.280–2.3 s). Example waveforms are shown in Fig. 1A.

All regular segments were therefore missing-fundamental stimuli within the passband 1–4 kHz, with the harmonics of orders of 4–16 and 50–200 most prominent for F0 = 250 Hz and F0 = 20 Hz segments, respectively. We deliberated whether to fix the harmonic orders or passband and opted for the latter to keep the signals most similar perceptually. The choice of adjusting the masker bandwidth to the F0 of the regular segment means that the spectrum of the noise segments in 250 Hz and 20 Hz trials had a slightly different spectral profile.

We presented regular segments between or around noise segments in order to isolate pitch (or regularity)-onset/offset responses from sound-onset/offset responses (Krumbholz et al., 2003). The idea stems from the observation that the canonical sound-onset response (i.e., N100m; Hari et al. (1987); Näätänen and Picton (1987)) “can be elicited by the onset of almost any kind of sound, irrespective of its physical or perceptual properties” (Krumbholz et al. (2003), pp. 765). That is, the presentation of a pitch-evoking sound in isolation would elicit not only a pitch-onset response (POR) but also an energy-onset response that is irrelevant to pitch. Critically, the POR has different electrophysiological characteristics (e.g., a later latency, a more anterior dipole location) than the N100m (Chait et al., 2005b; Gutschalk et al., 2004; Krishnan et al., 2012, 2014; Krumbholz et al., 2003; Ritter et al., 2005; Seither-Preisler et al., 2006). Moreover, the POR is absent if the repetition rate of the regular stimuli is below the lower limit of pitch (Gutschalk et al., 2004; Krumbholz et al., 2003; Ritter et al., 2005). Put differently, an increase of repetition rates did not evoke the POR if the increased repetition rate was still below 30 Hz, further demonstrating that the POR is different from the energy-onset response. To isolate the POR from the energy-onset response, the regular sounds were presented in stimulus sequences of Noise-Regular-Noise (NRN) or Regular-Noise-Regular (RNR) transitions (Fig. 1B; see Inline Supplementary Fig. S1 for cochleograms).

Each of 12 sequences (2 F0s x 3 stimulus types x 2 sequence types) was presented for 60 times over three sessions, leading to a total of 720 trials (12 stimuli x 60 repetitions) that were separated by an average inter-stimulus interval (ISIs) of 1000 ms (uniformly jittered in the range 900–1100 ms). Each of the 720 trials was created individually (i.e., the random noise and RIN in each trial was unique). Participants were instructed to listen attentively to the sounds and press a button at the end of each trial to aid alertness.

### 2.3 Data acquisition

MEG data were acquired with a 274-channel whole-head MEG scanner (CTF systems), using third-order axial gradiometers at a sampling rate of 1200 Hz. Three energized Head Position Indicator (HPI) coils were attached to fiducials (nasion, left and right preauricular) to ensure correct positioning within the dewar and to continuously monitor participants’ head movements. No additional head shape points were digitized at the time of recording. Stimuli were presented diotically at a comfortable listening level via flexible pneumatic tubes connected to piezo-electric transducers positioned approximately 1 m below the sensor array. Participants were instructed to keep their eyes open and to only blink, if necessary, in the ISI between trials.

In addition to the MEG data, standard magnetization-prepared rapid acquisition with gradient echo (MPRAGE) T1-weighted images were acquired using Siemens Allegra and Trio scanners at 3-Tesla for a subset of participants (*n* = 13).

### 2.4 Data analysis

MEG data were analyzed using MNE (Minimum Norm Current Estimates) Python package (v0.20.3; RRID:SCR_005972^3^), NoiseTools (v23-Jul-2020^4^), and custom codes in MATLAB and Python. MRI data were processed using FreeSurfer (v6.0.0; RRID:SCR_001847^5^) to generate cortical surface models to create boundary-element-models (BEMs) and source spaces.

#### 2.4.1 MEG data preprocessing

Using a Maxwell filter in MNE-Python (*mne.preprocessing.maxwell_filter*), external noise was projected out based on sensor geometry and temporal correlation (Taulu and Simola, 2006), while the recordings from all sessions were transformed to the first session based on the localization results (goodness of fit > 98%) of head position indicator (HPI) coils (mean head motion speed before Maxwell filtering = 1.34 ± 2.25 mm/s; after Maxwell filtering = 0.50 ± 0.45 mm/s). After high-pass filtering at 0.5 Hz using a Hamming windowed finite-impulse response (FIR) filter (one-pass, zero-phase) to remove slow drifts, and notch-stop filtering at 50, 100, …, 550 Hz to suppress power-line noise using a windowed FIR filter (two-pass, zero-phase), an independent component analysis (ICA) using the FASTICA algorithm extracted 40 independent components (ICs) using *mne.preprocessing.ICA*. To exclude channels with excessive readings from estimated ICs, a rejection criterion of 5000 fT/cm was used. Based on topographies and time-series, ICs dominated by artifacts such as eye-blinks, horizontal saccadic movements, and heartbeats were determined manually, then removed from the data. Subsequently, the continuous data were low-pass filtered at 40 Hz (Hamming windowed FIR, one-pass, zero-phase), down-sampled to 600 Hz, then epoched from −250 ms to +2550 ms with respect to stimulus onset (i.e., 250 ms before/after the stimulus onset/offset).

#### 2.4.2 Sensor-level analysis

For the evoked response analyses, a denoising source separation (DSS) algorithm (de Cheveigné and Simon, 2008) was applied to epoched (from −250 ms to +2550 ms after stimulus onset) trials of all sensors using *nt_dss1* in NoiseTools. The DSS algorithm finds orthonormal components maximizing a ‘bias’ function, which is defined by the evoked-to-total power ratio, to find activity that is reproducibly evoked by the stimulus. The first DSS component (DSS1) was chosen as the most reliably evoked component across trials. Because attenuation due to noise is removed by DSS, the signal-to-noise ratio of DSS1 is greater than the simple average if there exists any evoked activity in the signal (de Cheveigné and Simon, 2008). Since the signs of time-series can be arbitrary (the product of temporal and spatial weights equals to the original signal) in the DSS, polarities of the DSS1 components of all participants and conditions were flipped to match a component in a representative participant and condition (S02, HC250RNR). Note that the DSS transforms unaveraged trials, since it serves as a spatial filter (similar to PCA or ICA).

To study the evoked responses to pitch-onset/offset, the DSS1 trials were re-epoched from −200 ms to +900 ms after NR (Noise-to-Regular) and RN (Regular-to-Noise) transitions, and then averaged across trials for each of the six stimulus types. To study the evoked response to sound-onset, the DSS1 trials were re-epoched from −200 ms to +500 ms after stimulus-onset, and then averaged across trials for each of the six stimulus types with respect to regularity type (regular or noise).

#### 2.4.3 Source-level analysis

To construct individual source models, a T1-weighted MRI scan was fully automatically processed using recon-all in FreeSurfer, including intensity normalization, tissue segmentation, cortical surface extraction, spherical mapping, topological correction, and parcellation. For source space, 10,242 vertices on the ‘white surface’ (i.e., an interface between gray matter and white matter) were subsampled, of which spherical coordinates corresponded to the vertices of an icosahedron subdivided 5 times (ico-5), out of 120,000–150,000 vertices per hemisphere (*mne.setup_source_space*). For conductivity models, a boundary element model (BEM) of the inner skull surface (i.e., one-layer model) was created using a watershed algorithm (*mne.bem.make_watershed_bem*). For inverse operators, a loose constraint of 0.2 on dipole orientations and a weight of 0.8 on dipole depths were applied (Lin et al., 2006a, 2006b).

Since no head shape points were digitized at the time of MEG measurements, we manually identified the fiducial points on precise outer skin surfaces reconstructed from the T1-weighted images to align functional and anatomical spaces (i.e., coregistration of MEG and MRI data). Then, rigid-body transform matrices were estimated to align fiducial points in MEG and MRI data using a MNE-Python module (*mne.gui.coregistration*).

Epoching was done similarly to the sensor-level analysis: from −200 to +900 ms with respect to transitions, or from −200 to 500 ms with respect to sound-onset. To estimate source time courses on evoked responses (i.e., averaged time-locked responses), the noise-normalized MNE solution (dynamic statistical parametric mapping; dSPM) was used with a regularization parameter lambda of 0.12.

**Fig. 2:**
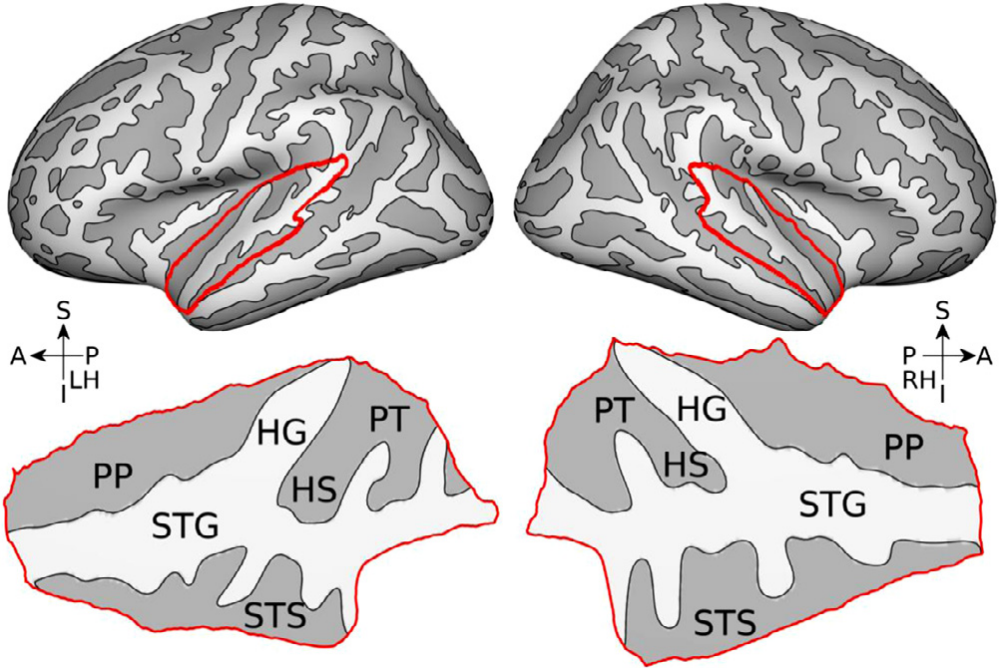
*Regions of interest.* Supratemporal planes on the cortical surface are marked in red. Binarized curvature is indicated in gray scale (brighter, convex; darker; concave). Orientations are indicated with arrows (A, anterior; P, posterior; S, superior; I, inferior; LH, left hemisphere; RH, right hemisphere). Landmarks on the supratemporal plane are marked (HG, Heschl's gyrus; HS, Heschl's sulcus; STG, superior temporal gyrus; STS, superior temporal sulcus; PP, planum polare; PT, planum temporale).

While the time courses of individual sources were estimated over the whole-cortex, we restricted our analysis to bilateral supratemporal planes (Fig. 2), as we did not find strong evoked responses elsewhere except for the perisylvian areas. The region of interest (ROI) was defined based on the Desikan-Killiany Atlas provided in FreeSurfer (regions labeled as ‘*transverse temporal*’ and ‘*superior temporal*’; left hemisphere: 510 vertices, 26.4 cm^2^; right hemisphere: 477 vertices, 25.39 cm^2^). For visualization of results, surface-mapped values (either dSPM or F-statistics) were nearest-neighbor interpolated on high-resolution template surfaces (‘*fsaverage*’; 163,842 vertices per hemisphere), slightly smoothed (2 iterations of neighbor averaging), and then projected onto flattened ROI surfaces.

#### 2.4.4 Statistical inference

Traditionally, the analysis of evoked responses involved extracting latencies and magnitudes from well-known stereotypical (“canonical”) peaks (Picton et al., 1974). However, identifying a peak (even from a visual inspection) entails a fallacy of circular reasoning (also known as “double-dipping”) as discussed in Luck and Gaspelin (2017). Moreover, this approach is reliable only when there exist well-defined peaks in all conditions, which was not the case in the current data (e.g., transitions to RIN20 did not evoke clear peaks) in line with previous studies (e.g., Krumbholz et al. (2003)). This motivated us to use massive univariate tests (i.e., sample-wise regression) to analyze effects.

At each temporal or spatiotemporal sample, all mixed effects were tested using multi-way repeated-measures ANOVA (RM-ANOVA) while accounting for between-subject variance. For transitions, two within-subject factors of Stimulus Type (HC, CT, or RIN) and Frequency (20 or 250 Hz) were tested. We were primarily interested in the following contrasts: (i) the main effect of Frequency on the NR transition (i.e., comparing regularity-onset responses for pitch-relevant regularity at 250 Hz vs. pitch-irrelevant regularity at 20 Hz), (ii) the interaction between Frequency and Stimulus Type on the NR transition (i.e., comparing pitch-related responses across various regularity types). For sound-onsets, three within-subject factors of Regularity (Noise or Regular), Stimulus Type (HC, CT, or RIN), and Frequency (20 or 250 Hz) were tested. We were mainly interested in (iii) the main effect of Regularity and two-way and three-way interactions with Regularity on sound onset.

For multiple comparison correction for massive univariate tests, a cluster-based permutation test (Maris and Oostenveld, 2007) with a cluster-forming threshold of *p* < 0.001 and 10,000 permutations was used as implemented in MNE-Python (*mne.stats.permutation_cluster_test* for sensor-level; *mne.stats.spatio_temporal_cluster_test* for source-level). The alpha level for the cluster-wise p-values was adjusted by Bonferroni-Holm correction for the number of contrasts tested in order to control the family-wise error rate (FWER) below 0.05 (Cramer et al., 2016).

### 2.5 Data and code availability

MEG and MRI data will be made available upon reasonable request. All MATLAB and Python code used to analyze data and create visualization for the current study is available on the Open Science Framework.^6^

## 3 Results

### 3.1 Sensor level analysis

**Fig. 3:**
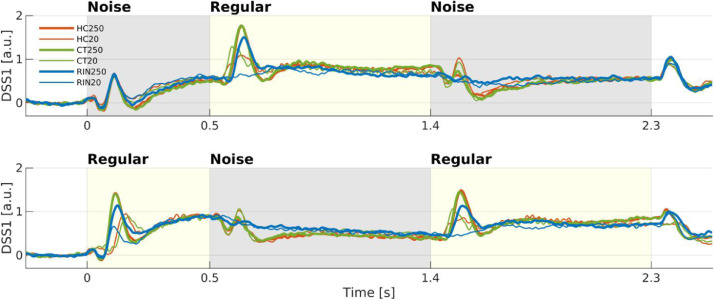
*Grand-average (n = 19) DSS1 component over the whole trial.* The evoked DSS1 components to Noise-Regular-Noise (upper) or Regular-Noise-Regular (lower) sequences are plotted for each Stimulus Type and Frequency (red, HC; green, CT; blue, RIN; thick, 250 Hz; thin, 20 Hz). Shading indicates the presence of sound (gray, Noise; yellow, Regular).

We first investigated the overall response to the entire trial as weighted linear sums of all sensors. The DSS analysis decomposed the data into components that are consistent, or replicable across trials (i.e., stimulus-evoked). The average ratio of the power of the evoked response compared to the overall power for the first DSS component (DSS1) was 43.27% ± 9.61%. The evoked response of the DSS1 over the whole NRN and NRN sequences is shown for the different trial types in Fig. 3.

#### 3.1.1 Evoked responses to NR vs. RN transitions reveal that the effect of pitch was similar across stimulus types

**Fig. 4:**
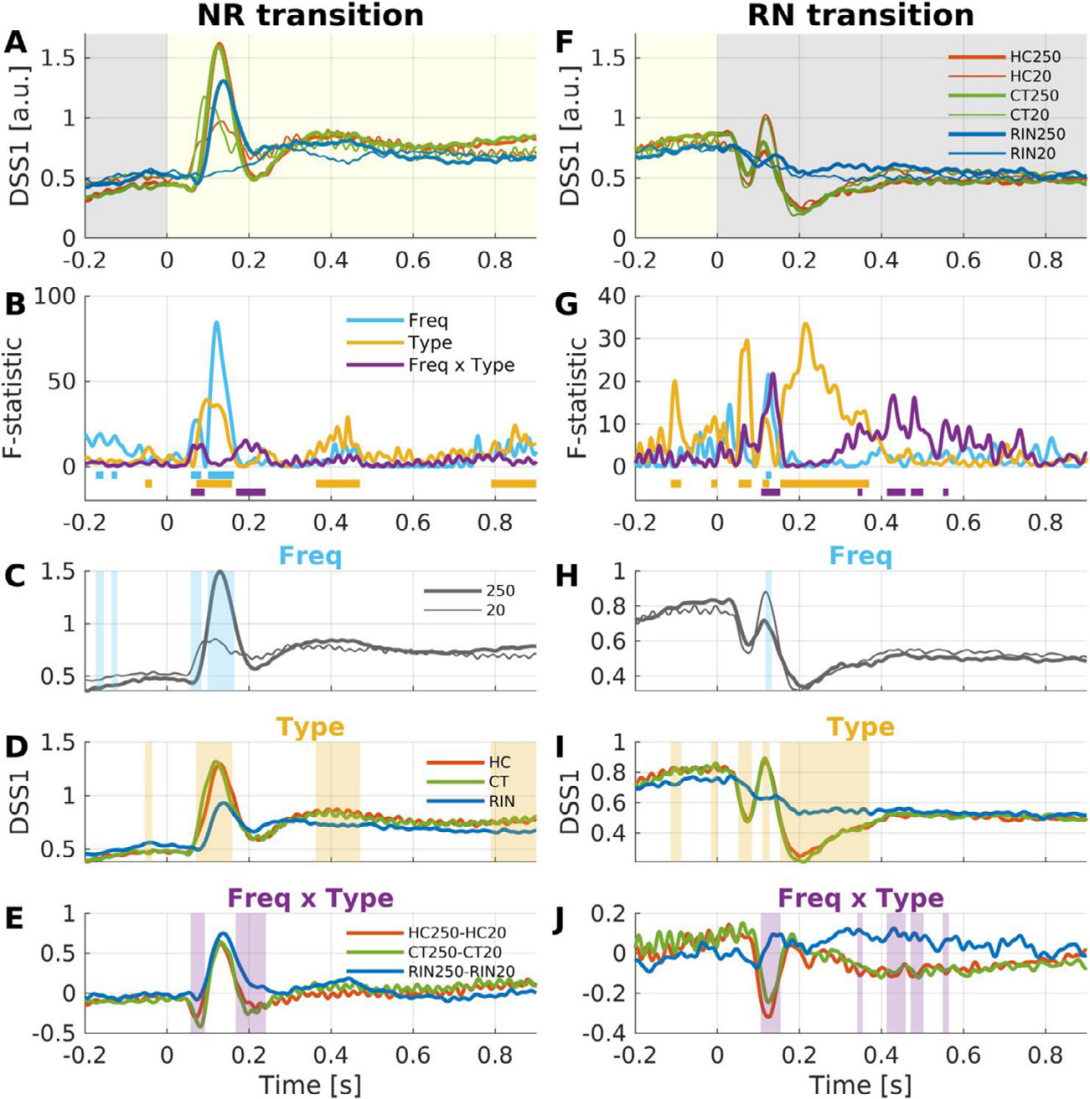
*Repeated-measures ANOVA on DSS1 response to transitions.* (A) Average (*n* = 19) DSS1 components for the NR transition for each condition (red, HC; green, CT; blue, RIN; thick, 250 Hz; thin, 20 Hz). (B) F-statistics over time for main effects and interaction (blue, Frequency; yellow, Stimulus Type; purple, Frequency x Stimulus Type). Significant intervals are marked by thick horizontal lines (FWER < 0.05). (C–E) Average DSS1 for the main effect of Frequency (C), Stimulus Type (D), and their interaction (E). Shading indicates where the effect is significant (see also B). The same scheme was used for RN transition (F–J).

We next investigated the evoked responses to the transitions between noise and regular segments. For this purpose, trials were re-epoched for intervals of [−0.2, +0.9] s relative to each transition: for NR transitions between 0.3–1.4 s after stimulus-onset in NRN, and between 1.2–2.3 s in RNR; for RN transitions between 1.2–2.3 s in NRN, and between 0.3–1.4 s in RNR. A RM-ANOVA with factors Stimulus Type (HC, CT, RIN) and Frequency (20 Hz, 250 Hz) revealed both main effects and an interaction (Fig. 4, Inline Supplementary Table S1).

Responses to NR transitions can be interpreted as regularity-onset responses while the overall sound energy remains constant. The 250-Hz regular stimuli evoke the percept of pitch and the 20-Hz regular stimuli do not. Therefore, evoked responses that are different between 250-Hz NR vs. 20-Hz NR transitions can be interpreted as an effect of pitch (within regular stimuli). The RM-ANOVA revealed a main effect of Frequency (i.e., averaged across all stimulus types), which was significant for intervals of [58, 83] ms and [100, 163] ms, showing a greater evoked response to 250 Hz than 20 Hz during the latter interval (Fig. 4C). Note that, while the peak amplitude for RIN250 was somewhat smaller than for HC250 and CT250 at around 130 ms after transition (Fig. 4A), this difference did not reach significance. That is, the effect of pitch at around 130 ms was statistically indistinguishable between pitch types. However, a main effect of Stimulus Type (i.e., averaging across 20 Hz and 250 Hz frequencies) revealed that RIN evoked significantly weaker responses compared to HC and CT for intervals of [72, 158], [363, 470], and, [790, 900] ms (Fig. 4D), which was presumably driven by the weak response to RIN20 (Fig. 4A). The interaction between Frequency and Stimulus Type tested whether the effect of pitch differed between pitch types; this was the case for time intervals [58, 92] and [168, 240] ms and was due to smaller response troughs for RIN (Fig. 4E).

In contrast to NR transitions, responses to RN transitions can be interpreted as regularity-offset responses. The main effect of Frequency was brief ([118, 132] ms), with a greater amplitude peak for 20 Hz (Fig. 4H). The main effect of Stimulus Type was significant over multiple intervals of [52, 83], [110, 127], and [153, 370] ms (Fig. 4I). The transition response from RIN to noise decayed more slowly, while the transition responses from HC and CT showed distinct negative-positive-negative deflections. The interaction between Frequency and Stimulus Type was also significant for intervals of [107, 153], [342, 353], [413, 458], [472, 502], and [550, 563] ms, which was due mainly to the greater deflections from HC20 and CT20 than from HC250 and CT250 (Fig. 4J).

Note that the pre-baseline effects (Frequency and Stimulus Type on NR; Stimulus Type on NR) were likely due to the fact that the epochs were baseline corrected with respect to the time period before stimulus onset ([−250, 0] ms), but not before each transition.

#### 3.1.2 Evoked responses to sound-onset reveal that the effect of pitch has an earlier latency than in NR transitions

**Fig. 5:**
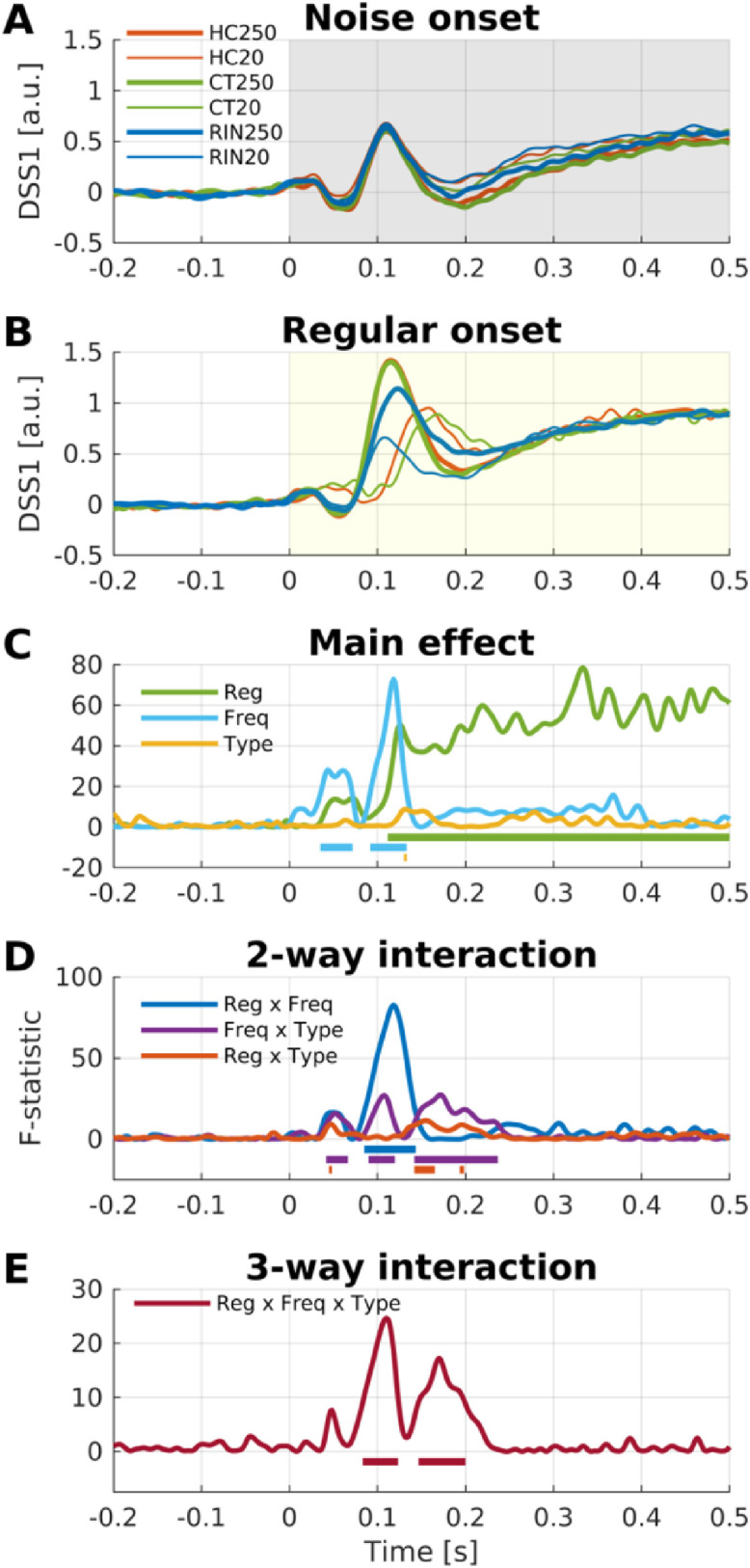
*Repeated-measures ANOVA on DSS1 response to onsets.* (A–B) Average (*n* = 19) DSS1 components for noise onset (A) and regular sound onset (B) for each condition. (C–E) F-statistics over time for main effects and interaction. Significant intervals are marked by thick horizontal lines (FWER < 0.05).

In addition, we also compared noise-sound-onset responses and regular-sound-onset responses, both of which are accompanied with energy-onset responses, to determine whether the pitch effect in sound-onset responses is similar or different from the pitch effect in regularity-onset responses described in the previous section. For this purpose, the trials were re-epoched to intervals of [−0.2, +0.5] s after stimulus-onset. A RM-ANOVA with factors Regularity (Noise-onset, Regular-onset), Stimulus Type, and Frequency revealed several main effects and interactions (Fig. 5, Inline Supplementary Table S2). The main effect of Regularity revealed that regular sounds evoked a stronger sustained response compared to noise sounds during [112, 500] ms after sound-onset (Fig. 5C). The main effect of Frequency was significant during the intervals of [35, 72] ms and [92, 133] ms after sound-onset, where 250 Hz stimuli evoked greater responses than 20 Hz stimuli. The main effect of Frequency, particularly around 100 ms, was driven by responses to regular segments, as highlighted by the three-way interaction ([83, 123] ms; Fig. 5E). The effect of pitch in regular stimuli was revealed via the two-way interaction between Regularity and Frequency ([85, 143] ms; Fig. 5D); note that this was earlier than the effect of pitch in NR transitions ([100, 163] ms; Fig. 4C).

### 3.2 Source level analysis

The source-level analyses in this section follow the same structure and logic as in Section 3.1. Inline Supplementary Movie S1 displays the estimated source time-courses to NR/RN transitions. Overall, bilateral activations over the HG, PT, PP, and posterior STS were found across the conditions. The NR transitions (Inline Supplementary Movie S1A–F) showed greater transition responses than the RN transitions (Inline Supplementary Movie S1E–J), except for RIN20, which showed very weak responses for both NR and RN transitions (Inline Supplementary Movie S1C, F).

Inline Supplementary Movie S2 shows the same as Inline Supplementary Movie S1, just for sound onset. The responses to noise-sound-onsets (Inline Supplementary Movie S2A–F) were weaker than to regular-sound-onsets (Inline Supplementary Movie S2G–L). Note that the actual stimuli for Inline Supplementary Movie S2A–C were identically filtered random noise segments, which is also the case for Inline Supplementary Movie S2D–F (see Methods).

#### 3.2.1 Sources of evoked responses to NR vs. RN transitions reveal that the effect of pitch is localized on Heschl's gyrus and planum temporale

**Fig. 6:**
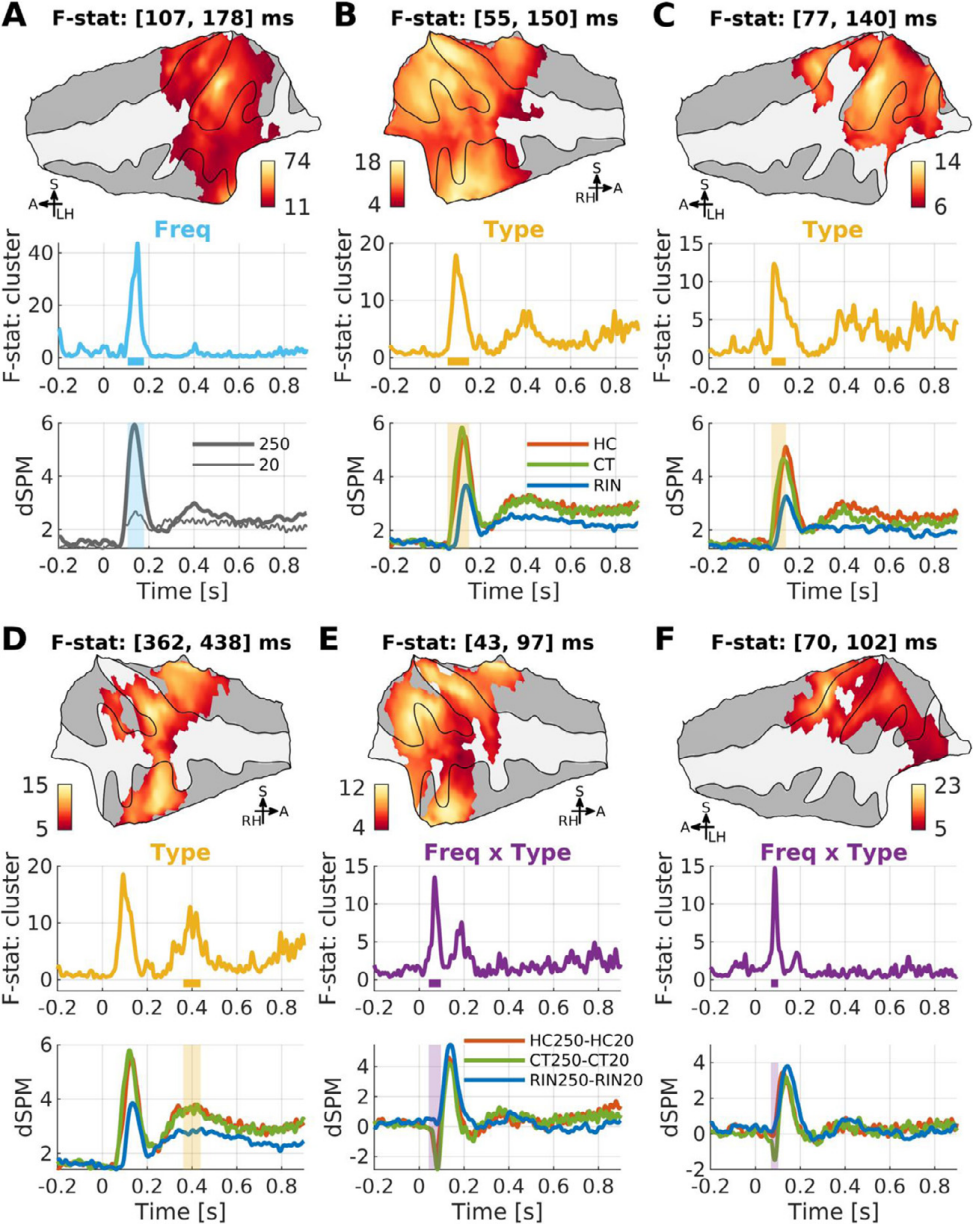
*Repeated-measures ANOVAs on source time courses for NR transitions.* Time-averaged (*n* = 10) F-map projected on a flattened supratemporal plane (upper), vertex-averaged F-timeseries (middle), and vertex-averaged source time-course (dSPM, dynamic statistical parametric mapping; lower) for contrasting conditions are shown for each significant cluster (FWER < 0.05). F-maps are thresholded by the significant clusters. Tested effects are (A) main effect of Frequency (250 Hz vs. 20 Hz), (B–D) main effect of Stimulus Type (HC vs. CT vs. RIN), (E–F) interaction between Frequency and Stimulus Type. Please refer to Fig. 2 for anatomical landmarks.

A two-way RM-ANOVA with factors Stimulus Type and Frequency revealed significant main effects and an interaction for NR transitions (Fig. 6, Inline Supplementary Table S3). The main effect of Frequency was significant between [107, 178] ms after transition, most strongly in the anterior planum temporale and its vicinity including the posterior Heschl's gyrus in the left hemisphere. Regular stimuli with F0 = 250 Hz evoked greater responses than those with F0 = 20 Hz. While the responses to regular 250 Hz stimuli were strong in the right hemisphere, this was also the case for regular 20 Hz stimuli (particularly for CT20; see Inline Supplementary Movie S1). Thus, the effect of Frequency in the right hemisphere (localized in the planum polare) was not significant after correction (FWER > 0.05): cluster-wise corrected-*p* = 0.0462 for a Bonferroni-Holm adjusted alpha of 0.0167 (Inline Supplementary Fig. S2). The main effect of Stimulus Type was significant bilaterally in an earlier period ([55, 150] ms in the right hemisphere (Fig. 6B); [77, 140] ms in the left hemisphere (Fig. 6C)) and a later phase ([362, 438] ms in the right hemisphere (Fig. 6D)) over large areas including the planum temporale, Heschl's gyrus, and lateral superior temporal gyrus. This effect was driven by weaker responses to RIN stimuli than HC and CT stimuli in all clusters. The interaction between Frequency and Stimulus Type was significant in an early phase ([43, 97] ms in the right hemisphere (Fig. 6E); [70, 102] ms in the left hemisphere (Fig. 6F)) over the planum temporale and Heschl's gyrus, which was due to RIN250 and RIN20 evoking similar responses. The negative peaks for HC250-HC20 and CT250-CT20 were due to earlier rises to CT20 and HC20 compared to CT250 and HC250 during the early phase. No significant effect was found for RN transitions (FWER > 0.05).

#### 3.2.2 Sources of evoked responses to sound onsets reveal that the effect of regularity was localized in the planum temporale and superior temporal sulcus

**Fig. 7:**
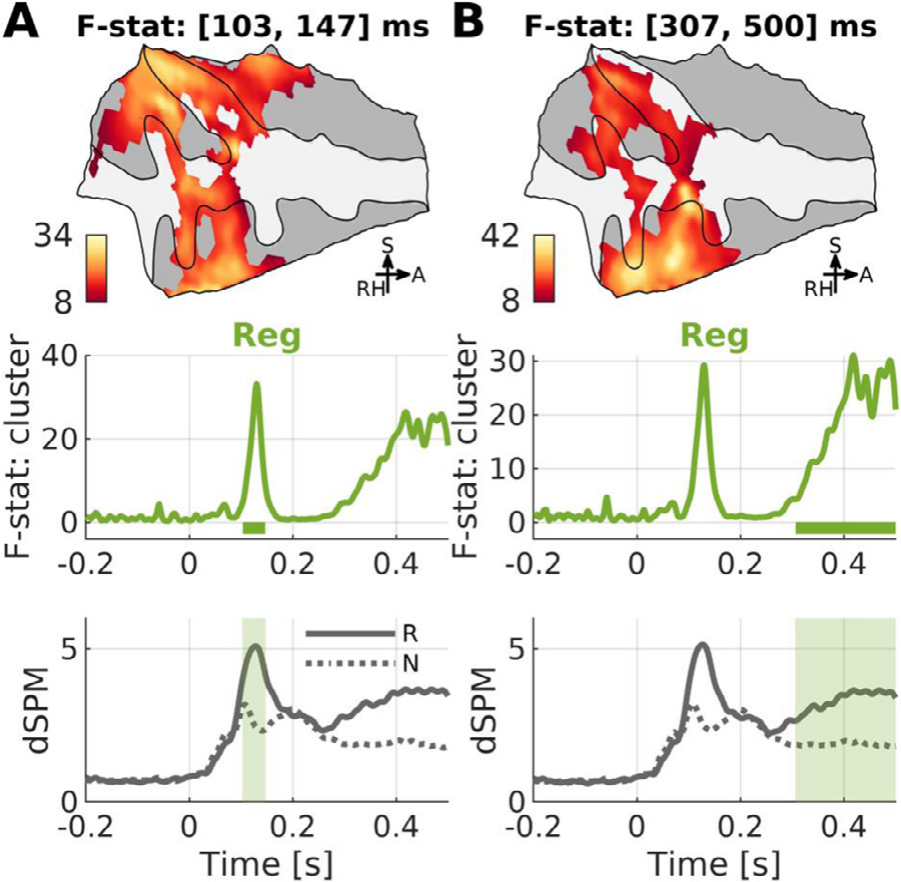
*Main effect of Regularity (regular vs. noise).* Time-averaged (*n* = 10) F-map projected on a flattened supratemporal plane (upper), vertex-averaged F-timeseries (middle), and vertex-averaged source time course (dSPM, dynamic statistical parametric mapping; lower) for contrasting conditions are shown for each significant cluster (FWER < 0.05). F-maps are thresholded by the significant clusters. Please refer to Fig. 2 for anatomical landmarks.

A three-way RM-ANOVA with factors Regularity, Stimulus Type, and Frequency revealed significant main effects for Frequency and Regularity on onset responses. The main effect of Regularity (i.e., greater response to regular stimuli compared to noise stimuli) was significant transiently during [103, 147] ms after sound-onset and in a sustained interval ([307, 500] ms) over the right PT (Fig. 7, Inline Supplementary Table S4). The activation during the later phase was stronger in the STS.

## 4 Discussion

The results demonstrate a profound sensitivity to pitch information at the level of human auditory cortex. Transitions from noise to pitch-relevant regularity (F0 = 250 Hz) evoked larger responses than the ones to pitch-irrelevant regularity (F0 = 20 Hz), at both sensor and source levels. Transitions from pitch-relevant regularity to noise also evoked a greater response than from pitch-irrelevant regularity at the sensor level. Additionally, regular sound onsets evoked sustained responses compared to noise onsets.

### 4.1 Invariant pitch extractor

Various forms of regularity can evoke a pitch percept (Chait et al., 2005a; Watkinson et al., 2005). Invariance with respect to various forms of regularity has been suggested as one of the criteria for the presumed “pitch center” or “pitch extractor” (de Cheveigné (2006); Hall and Plack (2009); though note that the necessity of such a strict invariance criterion has been questioned, Griffiths et al. (2010)). In the present study, the use of HC, CT, and RIN enabled us to explore the idea of invariance further. Moreover, the choice of repetition rates of 20 Hz and 250 Hz allowed us to disambiguate responses that can be attributed to the encoding of sound regularity in general from those that specifically encode pitch-relevant regularity (Krumbholz et al., 2003; Pressnitzer et al., 2001).

Pitch-relevant regularity evoked a greater response than pitch-irrelevant regularity during [107, 178] ms following the noise-to-regular transition (the effect of frequency; Fig. 6A). The corresponding cluster encompassed all of HG, PT and the lateral STG adjacent to PT without any spatiotemporal overlap with a cluster for the interaction between frequency and stimulus type (i.e., peaking in the first transverse sulcus, which is the anterior boundary of the transverse gyrus, during [70, 102] ms after noise-to-regular transition; Fig. 6F). That is, the effect of pitch was found without any difference across stimulus types, which is in support of the invariance hypothesis.

To better appreciate the anatomy of the cluster, we rendered the F-map for the effect of frequency on responses to noise-to-regular transitions on the outer (‘pial’) cortical surface (Inline Supplementary Fig. S3). This revealed that the maximal effect was localized along Heschl's sulcus. This is in agreement with recent human neurophysiological data showing pitch-related high gamma (70–120 Hz) responses in the lateral temporal convexity around the transverse temporal sulcus (i.e., Heschl's sulcus; *Fig. 5* in Gander et al. (2019)). While this study (Gander et al., 2019) only tested RIN stimuli, we show pitch-related responses in Heschl's sulcus for HC, CT, and RIN stimulus types. These results position posterior Heschl's sulcus as a putative “invariant pitch extractor”. Note that posterior Heschl's sulcus is broadly compatible with both previous results suggesting such a role for lateral HG (Griffiths, 2005; Patterson et al., 2002; Penagos et al., 2004), or antero-lateral PT (Barker et al., 2012).

The 20-Hz (i.e., pitch-irrelevant) regularity-onsets evoked weak responses to the 250-Hz regularity-onsets (Fig. 6A), which might raise the question whether the latter is indeed exclusive to pitch-relevant regularity. However, upon closer examination (Inline Supplementary Fig. S4 and Movie S1), it is apparent that the response to HC20 and CT20 peaked at around 90 ms after transition while RIN20 did not evoked any appreciable responses. In contrast, the 250-Hz regularity-onsets evoked responses that peaked at around 130–140 ms. The early peaks to 20 Hz and the later peaks to 250 Hz were detected in the DSS1 results (Fig. 4C). In addition, the spatial distributions of source activity (Inline Supplementary Fig. S4) suggest differential contributions of neuronal populations to each peak (though note that the massive univariate testing did not allow comparisons of activity between different latencies). Moreover, the absence of the early peak to RIN20 suggests that the early (∼90 ms) response might indeed reflect sensitivity to changes in acoustic energy (Hari et al., 1987; Lütkenhöner and Steinsträter, 1998), which were prominent for CT20 and HC20 but absent for RIN20 (see Inline Supplementary Fig. S1 for cochleograms).

### 4.2 Sound-onset vs. pitch-onset

Krumbholz and colleagues reported dipole fitting results in the left hemisphere of a single subject where the “pitch-onset response” (POR) dipoles were more anterior and inferior to the sound-onset response (labelled as “N100m” in the study) dipoles (Krumbholz et al., 2003; Lütkenhoner et al., 2011). The location of the POR appeared to be in HG (rather than PT) and was similar to known dipole locations of the P200m, but with different dipole orientations (Lütkenhöner and Steinsträter, 1998).

Krumbholz et al. (2003) found similar early (∼100 ms) responses to both the silence-to-noise and silence-to-RIN conditions. In contrast, we found a significant effect of regularity on sound-onset responses (i.e., responses to regular-sound-onset being greater than to noise-sound-onset) at both sensor and source levels (Inline Supplementary Movie S2L). In the source-level analysis, the regular-sound-onset evoked greater transient ([103, 147] ms) and sustained ([307, 500] ms) responses than the noise-sound-onset in the medial HG/PT and the superior temporal sulcus, respectively (Fig. 7), indicating additional activation associated with regularity accompanying the sound-onset response. In the present study, HC and CT were used in addition to RIN, whereas previous studies mostly focused on RIN. However, the differential onset response between noise and pitch cannot simply be explained by the stimulus type because we found no interaction between regularity and stimulus type. Thus, different processing methods (i.e., DSS, MNE) and a greater number of participants (i.e., *n* = 20 vs. *n* = 8) might account for this difference in results.

Specifically for HC and CT, when the regularity evoked a pitch (Inline Supplementary Movie S2J and Movie S2K), the sound-onset responses appear to be a superposition of pitch-onset response (Inline Supplementary Movie S1D and Movie S1E) and noise-onset response (Inline Supplementary Movie S2D and Movie S2E), with a delay of about 10 ms (peak latencies: 120/117 ms for HC250/CT250-sound-onset; 133/128 ms for HC250/CT250-pitch-onset). In the case of pitch-irrelevant regularity (20 Hz), regular-sound-onset responses appear to be a superposition of regularity-onset responses (Inline Supplementary Movie S1A and Movie S1B) that are delayed by about 22/74 ms (peak latencies: 160/167 for HC20/CT20-sound-onset; 138/93 ms for HC20/CT20-regularity-onset); this can be explained by the fact that the initial cosine ramp of 20 ms attenuated the first click (or harmonic complex) that was followed by the second click after 50 ms. However, this idea of a (linear) superposition of sound-onset and pitch-onset responses needs to be explored more systematically in future studies.

### 4.3 Idiosyncrasy of RIN

As discussed earlier, responses to RIN were different from HC and CT at multiple levels. In particular, regularity-onset responses were generally weaker for RIN than HC and CT, which was driven by the absence of an evoked response for RIN with F0 of 20 Hz. One possible explanation is the weaker pitch salience of RIN stimuli compared to HC and CT stimuli. The 16 iterations used in the current study have been shown to evoke a salient pitch percept in previous studies (Hall and Plack, 2009; Krishnan et al., 2010; Lütkenhöner et al., 2005), while additional gains in pitch discriminability (i.e., the difference limen of frequency) and neural pitch encoding strength (i.e., autocorrelation at the delay of 1/F0 in the frequency-following response) decay after about 12 iterations (Krishnan et al., 2010). However, it is unclear whether pitch salience might explain the RIN-specific weaker responses in the current data, as BOLD responses in the lateral HG have previously been found to be indifferent across various periodic stimuli with varying degrees of pitch salience (Hall and Plack, 2009). Another possible explanation for the greater time-locked responses to HC and CT stimuli is that the inter-trial phase synchrony was larger for HC and CT stimuli than for RIN stimuli. While all stimuli were unique across trials, this inter-trial phase randomization might have had the greatest effect for RIN stimuli.

As mentioned earlier, Barker et al. (2012) raised the concern that slow spectrotemporal modulations inherent in RIN might drive neural activation in HG, which could indeed be problematic when comparing responses to RIN with responses to static random noise. However, as shown in previous human electrophysiological studies (Gander et al., 2019; Griffiths et al., 2010), a comparison between responses to pitch-evoking RIN and non-pitch-evoking RIN replicated the main findings of high-gamma oscillations for pitch-evoking RIN over HG and PT. This could not have been the case if the response was solely driven by slow spectrotemporal modulations, since both pitch-evoking and non-pitch-evoking RIN comprised such modulations. In the current study, we also compared responses to pitch-relevant regular sounds (250 Hz) and pitch-irrelevant regular sounds (20 Hz) to cancel responses associated with regularity. Thus, the unique responses to pitch-evoking RIN cannot be attributed to inherent spectrotemporal modulations.

### 4.4 Regularity-offset response

Both at the sensor and source levels, we observed regularity-offset responses from regular-to-noise transitions (Fig. 3 and Inline Supplementary Movie S1). This offset response was greater for pitch-relevant regularity compared to pitch-irrelevant regularity for a brief period ([118, 132] ms after pitch-offset) at the sensor level (Fig. 4H). Furthermore, this pitch-offset response was different across stimulus types: it was essentially absent for RIN stimuli, but robust for HC and CT stimuli.

In a previous study (Krumbholz et al., 2003), a transition from pitch-evoking RIN to random noise did not evoke prominent MEG responses, which is in line with the current data also demonstrating an asymmetry in regularity onset/offset responses for RIN stimuli. That is, RIN-to-noise transitions evoked a very weak or no response. A later, highly powered (6680 trials for one condition) MEG study showed that the regular-to-noise transition evoked a monotonic positive deflection, which seemingly reflected the decay of a sustained response to regularity, rather than the onset of noise (Lütkenhoner et al., 2011). In our data, such a decaying process was also observed for pitch-irrelevant RIN (i.e., F0 = 20 Hz), suggesting that this process might be related to regularity rather than specifically to pitch. Another interesting point is that the regular-to-noise transition from RIN250 only evoked a small positive deflection (Inline Supplementary Movie S1J), unlike the RIN20 only with a monotonous decay (Inline Supplementary Movie S1G), at the source level in contrast to the sensor-level analyses in the previous studies (Krumbholz et al., 2003; Lütkenhoner et al., 2011); this suggests a transient pitch-offset response. It is possible that this difference between transitions from RIN250 and RIN20 could be attributed to the properties of the subsequent noise segment, which were filtered slightly differently for the 250 Hz and 20 Hz trials (see Methods). However, the silence-to-noise responses were virtually equivalent between the RIN20 and RIN250 sequences (Inline Supplementary Movie S2C and Movie S2F), rendering such an explanation unlikely.

CT stimuli evoke a pitch-offset response in stimuli where the clicks were presented with a repetition rate that was accelerated up to the target F0 and then deaccelerated (Lütkenhöner et al., 2005). In our data, although weaker than onset-responses, offset-responses were observed after regular-to-noise transitions for both CT and HC stimuli (Fig. 3). This response was greater after HC20 and CT20 than HC250 and CT250, and seemed similar to noise-onset responses. One possible explanation is that, when the regular stimulus is temporally sparser than the noise stimulus (which is the case for CT and HC compared to RIN, and even more so when F0 = 20 Hz), the regular-to-noise transition results in a contrast in short-term (1/20 Hz^−1^ = 50 ms) spectral energy (see Inline Supplementary Fig. S1 for cochleograms), and thereby evokes a noise-onset-like response on top of the decaying response to regularity.

### 4.5 Limitations

In this section, we discuss some methodological limitations with respect to the interpretation and generalization of the results.

#### 4.5.1 Alternative explanation of frequency effect

The current study is based on only two F0s, one well below and the other well above the lower limit of pitch. While this selection was made for practical reasons, it does not allow comparison of responses to different pitch-relevant (or pitch-irrelevant) F0s. One alternative explanation of the greater response to 250 Hz than 20 Hz could therefore be that it merely reflects the higher stimulation rate. However, we do not think this interpretation applies here. First, it is well established that the human auditory cortex prefers low rates (< 10 Hz) of amplitude modulation (Joris et al., 2004; Kim et al., 2020; Overath et al., 2012; Wang et al., 2012), which might be linked to the universal peak syllabic rate of ∼7 Hz in various human languages (Ding et al., 2017). Therefore, a rapid repetition rate of 250 Hz is unlikely to evoke greater phase-locked responses than 20 Hz, and Inline Supplementary Fig. S5 shows that this was indeed not the case in the current data. Second, previous studies have established that increasing the repetition rate below 30 Hz does not lead to an increase in the amplitude of the POR (Krumbholz et al. (2003); Lütkenhöner et al. (2005); see also Wang et al. (2012)).

Another explanation could be that the masking noise in 250 Hz regular stimuli (over 125–375 Hz) would have induced stronger responses while that in 20 Hz regular stimuli (over 10–30 Hz) was preferentially attenuated during the presentation via the pneumatic tube. This possibility, however, can be easily ruled out by the fact that the noise segments that were equally filtered and masked (thus with the same energy difference) did not evoke different responses (Fig. 5A).

#### 4.5.2 Transient response to pitch onset

The current study mainly focused on evoked responses to sounds with pitch-evoking regularity, known as the POR (Krumbholz et al., 2003). This raises the question how a transient evoked response like the POR is related to the sustained percept of pitch. Note that our argument of an “invariant pitch extractor” does not reflect the viewpoint that the transient response is the neural representation of pitch *per se*. A more nuanced interpretation of the current findings is that the F0 effect on evoked responses reflects cortical processes that detects changes in pitch salience (or autocorrelation structures within a certain repetition rate range). Induced responses (in particular the gamma oscillations) have been suggested as a potential neural correlate of a sustained percept of pitch. For example, electrophysiological data recorded in human auditory cortex and macaque auditory cortex (Gander et al., 2019; Griffiths et al., 2010; Kikuchi et al., 2019) revealed high gamma (80–120 Hz) oscillations starting about 70 ms after pitch onset that persisted throughout the pitch sound. A human MEG study (Sedley et al., 2012) also identified a strong gamma band (70–140 Hz) oscillation from “a virtual electrode” projection during a pitch-evoking RIN sound.

We suggest that the evoked responses to pitch onset can be explained as a characteristic signal of pitch extraction in terms of predictive coding (Friston and Kiebel, 2009). Friston (2005) showed that an evoked cortical response accompanied with an extra classical receptive fields effect (i.e., the modulation of receptive field properties by backward and lateral connections) can be regarded as a failure of the suppression of a prediction error. In this view (Friston, 2005), an event-related potential/field, such as the N100m, is interpreted as an unsuppressed alpha oscillation due to “a violation of statistical regularities that have been learned” from preceding sensory inputs. Indeed, using dynamic causal modeling (DCM) in human electrophysiological recordings, Kumar et al. (2011) revealed the modulation of connectivity amongst subregions of the HG, which predicted evoked responses to RINs. Specifically, the strengths of backward connections from lateral to medial and middle subregions of HG were increased as a function of pitch salience (i.e., the number of iterations of RIN). This finding suggests that the F0 effect on the evoked response reflects a prediction error on pitch salience (i.e., an emergence of a specific autocorrelation structure while no particular structure is predicted) during the process of pitch extraction, rather than a sustained representation of the pitch.

#### 4.5.3 Temporal coding

Given that the pitch of F0 was induced only by temporal cues in the current study, one could ask how temporal regularity encoded in evoked responses is related to pitch perception. The temporal coding of regularity is already visible in the evoked responses to 20-Hz regular stimuli after ∼200 ms post-transition (e.g., the thin line in Fig. 4C; the thin line in Fig. 6A). Autocorrelation computed on evoked responses (without low-pass filtering at 40 Hz) displayed peaks only at the 1/20 Hz^−1^ lag (and its harmonic 1/40 Hz^−1^) in HC20 and CT20 (more strongly in CT20) but not in RIN20. Also, no peak at 1/250 Hz^−1^ was found in any 250-Hz regular stimuli (Inline Supplementary Fig. S5). The temporal coding of 20 Hz (and 40 Hz) seems to be relevant to auditory stead-state responses (Galambos et al., 1981), which is known to be strongly induced by 40-Hz amplitude modulation. However, no temporal regularity was present in responses to pitch-evoking regular stimuli (250 Hz). This is in line with human electrophysiological data (Gander et al., 2019), where the evoked responses were found across a wide range of repetition rates, especially strongly in rates below the lower limit of pitch.

#### 4.5.4 Limits in the interpretation of source localization

MEG source-reconstruction should be interpreted with appropriate caution. Even with current state-of-the-art algorithms, the ill-posedness of the inverse problem marks inherent problems for source localization. Compared to EEG inverse solution, MEG solution is known to have fewer false positives and more false negatives due to its sensitivity to source orientation even with a similar number of sensors (Ahlfors et al., 2010). The orientation and depth of sources also play an important role in detectability (Hillebrand and Barnes, 2002). Nevertheless, the source-level analyses employed here are still meaningful since the same inverse solver was used for all conditions, thereby minimizing the possibility that the difference between conditions could have been driven by localization error.

#### 4.5.5 Limits in the interpretation of cluster-based permutation tests

We used a cluster-based permutation test (Maris and Oostenveld, 2007) on sample-wise regression to infer the significance of effects. That is, the inference was calculated for clusters, not for individual samples (e.g., time-points or vertices). We decided not to use sample-level inference because of its assumption of correspondence across samples. That is, a slight temporal misalignment across conditions could lead to invalid comparisons when using sample-level inference. In contrast, cluster-level inference provides a useful remedy for this problem, since it remains sensitive to differences between conditions even if samples are misaligned, albeit at the cost of precision. Therefore, it is important to note that cluster-level inference methods should not be interpreted at the level of a single time-point or a single vertex, but of a set of them (see also Sassenhagen and Draschkow (2019)).

## 5 Conclusions

In the current study, evoked responses specific to pitch-evoking regularity were found across various types of regular sounds in the supratemporal plane around Heschl's sulcus. While different regular stimuli evoked different responses reflecting their differences in acoustic properties, this difference was dissociated from a pure pitch-related response, suggesting a cortical correlate for an invariant pitch extractor.

## Data and code availability

MEG and MRI data can be available upon reasonable requests. All MATLAB and Python code used to analyze data and create visualization for the current study is available on the Open Science Framework (https://osf.io/kzjcd/).

## CRediT authorship contribution statement

**Seung-Goo Kim:** Methodology, Software, Formal analysis, Visualization, Writing – original draft. **Tobias Overath:** Conceptualization, Methodology, Investigation, Supervision, Writing – original draft. **William Sedley:** Conceptualization, Investigation, Writing – review & editing. **Sukhbinder Kumar:** Conceptualization, Investigation, Writing – review & editing. **Sundeep Teki:** Investigation, Writing – review & editing. **Yukiko Kikuchi:** Conceptualization, Writing – review & editing. **Roy Patterson:** Conceptualization, Writing – review & editing. **Timothy D. Griffiths:** Conceptualization, Resources, Supervision, Funding acquisition, Writing – review & editing.

## Appendix Supplementary materials

*Movie S1. Grand-averaged (n = 10) source time-courses for transition responses.* Source activity maps (top) and source time-courses (bottom) are shown for all conditions from 0 to 600 ms post-transition with 10 ms increment. Each source time-course line corresponds to a vertex on the supratemporal planes in the left (blue) and right (brown) hemispheres. The color scale is arbitrary thresholded between 0 and 10 (in dSPM arbitrary unit) and discretized to enhance visualization of isocontours. Please refer to the Results section for statistical inference on difference between conditions. Abbreviations: HC, Harmonic complex, CT, click train; RIN, regular interval noise; 20, F0 = 20 Hz; 250, F0 = 250 Hz; NR, noise-to-regular transition; RN, regular-to-noise transition; dSPM, dynamic statistical parametric mapping in arbitrary unit; LH, left hemisphere; RH, right hemisphere; HG, Heschl's gyrus; HS, Heschl's sulcus; STG, superior temporal gyrus; STS, superior temporal sulcus; PP, planum polare; PT, planum temporale. Image, application 1

*Movie S2. Grand-averaged (n = 10) source time-courses for sound-onset-responses.* Source activity maps (top) and source time-courses (bottom) are shown for all conditions from 0 to 500 ms post-transition with 10 ms increment. Each source time-course line corresponds to a vertex on the supratemporal planes in the left (blue) and right (brown) hemispheres. The color scale is arbitrary thresholded between 0 and 10 (in dSPM arbitrary unit) and discretized to enhance visualization of isocontours. Please refer to the Results section for statistical inference on difference between conditions. Abbreviations: HC, Harmonic complex, CT, click train; RIN, regular interval noise; 20, F0 = 20 Hz; 250, F0 = 250 Hz; NR, noise-to-regular transition; RN, regular-to-noise transition; dSPM, dynamic statistical parametric mapping in arbitrary unit; LH, left hemisphere; RH, right hemisphere; HG, Heschl's gyrus; HS, Heschl's sulcus; STG, superior temporal gyrus; STS, superior temporal sulcus; PP, planum polare; PT, planum temporale. Image, image 2

Image, image 3

## Footnotes

1 http://www.mathworks.com/products/matlab/

2 https://ccrma.stanford.edu/∼jos/sasp/Example_Synthesis_1_F_Noise.html

3 https://mne.tools/stable/index.html

4 http://audition.ens.fr/adc/NoiseTools/

5 http://freesurfer.net/

6 https://osf.io/kzjcd/

Supplementary material associated with this article can be found, in the online version, at doi:10.1016/j.neuroimage.2022.118879.
