# Supplementary material for: MEG correlates of temporal regularity relevant to pitch perception in human auditory cortex
Source: Neuroimage. 2022 Apr 1;249:118879. doi: 10.1016/j.neuroimage.2022.118879 (PMC8883111; doi:10.1016/j.neuroimage.2022.118879)
Supplement: Supplementary file 1 — Movie S1. Grand-averaged (n = 10) source time-courses for transition responses. Source activity maps (top) and source time-courses (bottom) are shown for all conditions from 0 to 600 ms post-transition with 10 ms increment. Each source time-course line corresponds to a vertex on the supratemporal planes in the left (blue) and right (brown) hemispheres. The color scale is arbitrary thresholded between 0 and 10 (in dSPM arbitrary unit) and discretized to enhance visualization of isocontours. Please refer to the Results section for statistical inference on difference between conditions. Abbreviations: HC, Harmonic complex, CT, click train; RIN, regular interval noise; 20, F0 = 20 Hz; 250, F0 = 250 Hz; NR, noise-to-regular transition; RN, regular-to-noise transition; dSPM, dynamic statistical parametric mapping in arbitrary unit; LH, left hemisphere; RH, right hemisphere; HG, Heschl's gyrus; HS, Heschl's sulcus; STG, superior temporal gyrus; STS, superior temporal sulcus; PP, planum polare; PT, planum temporale. [file mmc1.pdf]

Supplementary materials

Manuscript Title: MEG Correlates of Temporal Regularity Relevant to Pitch Perception in Human Auditory Cortex

Authors: Seung-Goo Kim, Ph.D.*, Tobias Overath, Ph.D.*; William Sedley, Ph.D.; Sukhbinder Kumar, Ph.D.; Sundeep Teki, Ph.D., Yukiko Kikuchi, Ph.D.; Roy Patterson, Ph.D.; Timothy D. Griffiths, M.D., Ph.D.

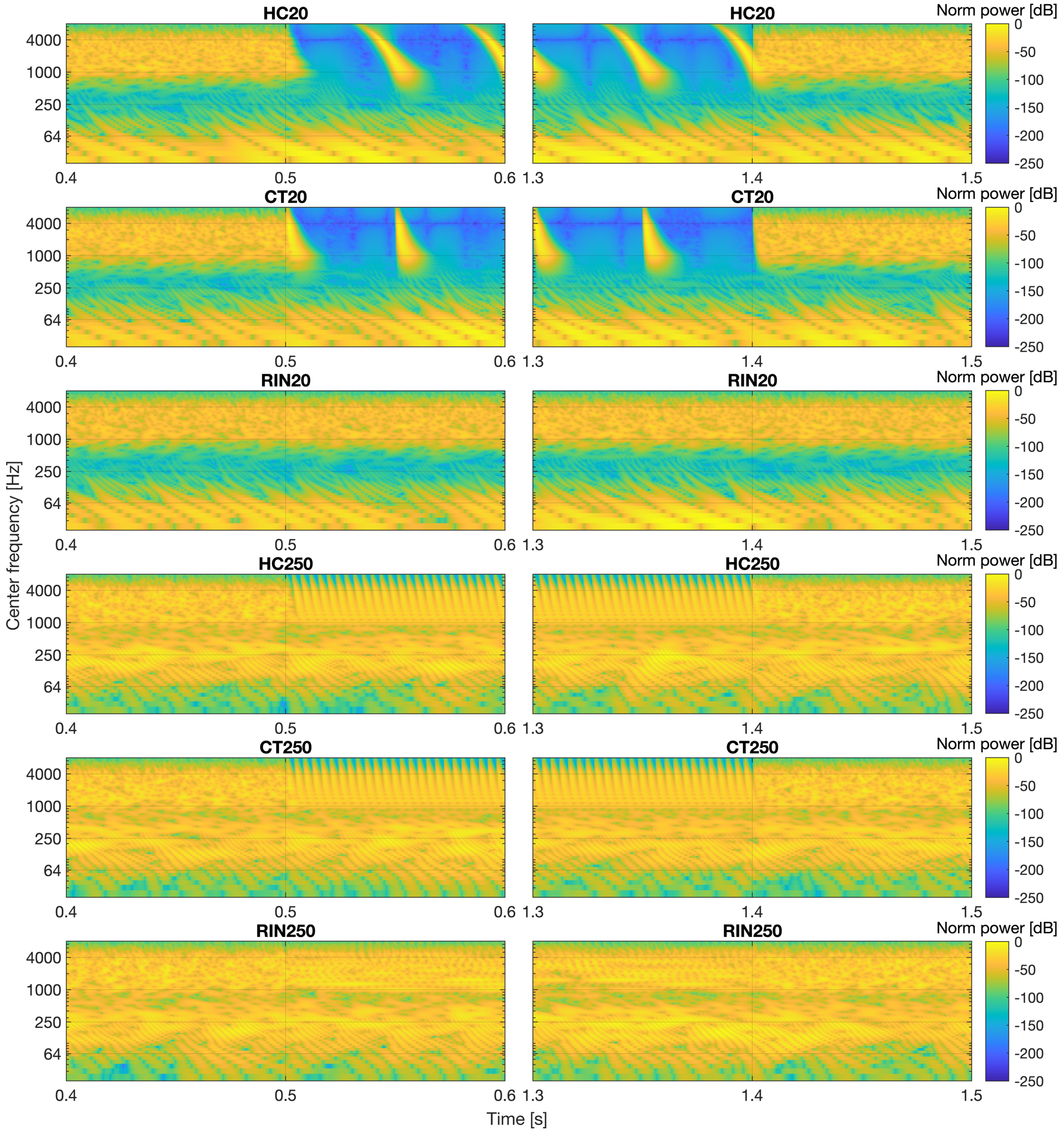

Figure S1. Cochleograms of regular-to-noise transitions. A gammatone filter bank with 64 center frequencies from 20 Hz to 8 kHz spaced in equivalent rectangular bandwidth (ERB) scale as implemented in a MATLAB function gammatoneFilterBank was used to simulate cochlear excitation over time in response to noise-to-regular-to-noise sequences with various regular stimuli (HC, harmonic complex; CT, click trains; RIN, regular interval noise) and two F0s (20 Hz and 250 Hz). Abscissae are scaled to ± 100 ms around noise-to-regular (0.5 s after stimulus onset; left) and regular-to-noise (1.4 s after stimulus onset; right) transitions for magnification. The length of a time frame is 1 ms for visualization. Color scales are normalized with respect to the minimal energy.

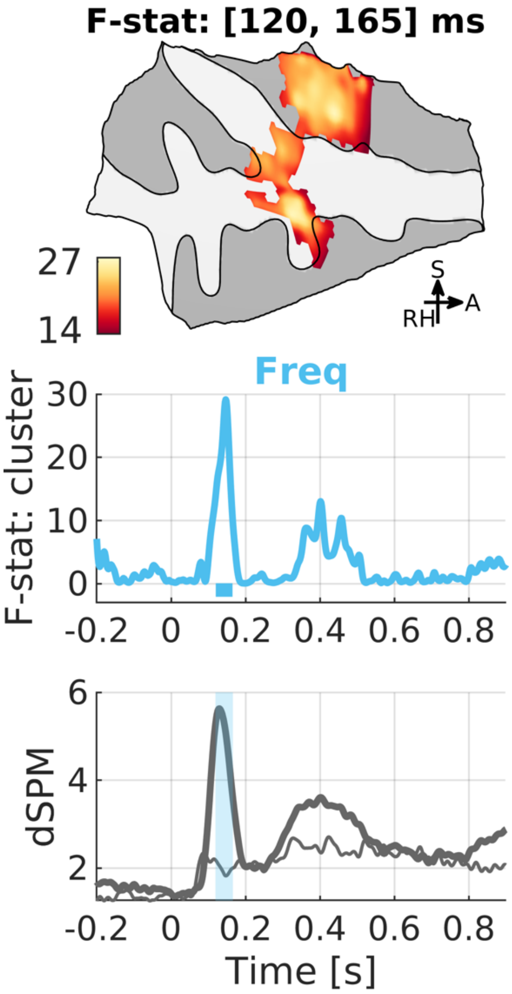

Figure S2. F-map for pitch-onset effect in the right hemisphere. Time-averaged F-map projected on a flattened supratemporal plane (top), vertex-averaged F-timeseries (middle), averaged source time-course (dSPM, dynamic statistical parametric mapping; bottom). The F-map (top) was thresholded at corrected-$p < 0.05$; note, though, that the cluster p-value was 0.0462, while Bonferroni-Holm correction for the number of contrasts adjusted the alpha to 0.0167 (FWER > 0.05).

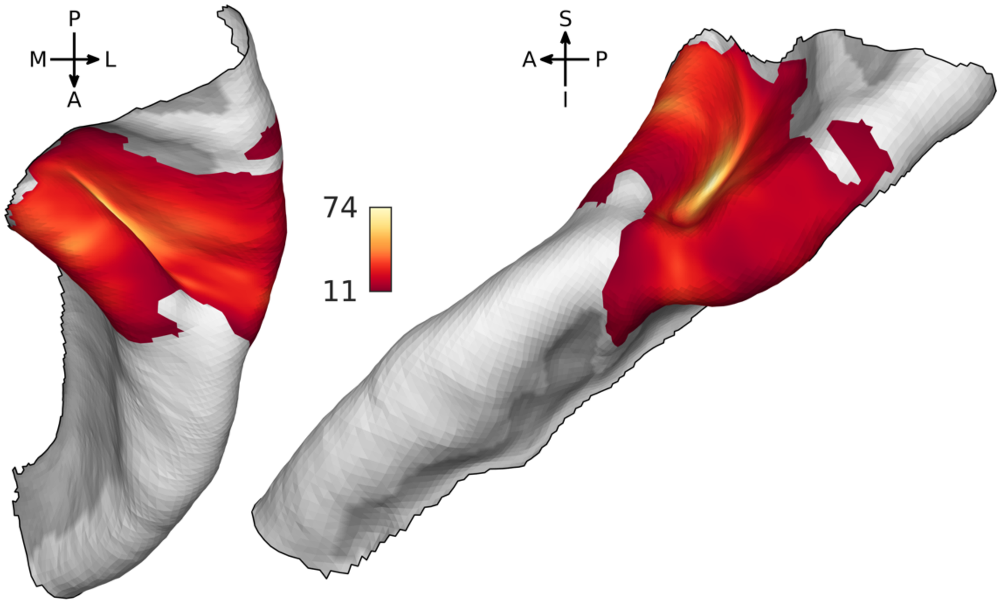

Figure S3. F-map for pitch effect on outer cortical surface. Time-averaged F-map for the effect of frequency on responses to noise-to-regular transition projected on the outer cortical surface of the left supratemporal plane (thresholded at FWER < 0.05). Orientations are marked by arrows (A, anterior; P, posterior; M, medial; L, lateral; S, superior; I, inferior).

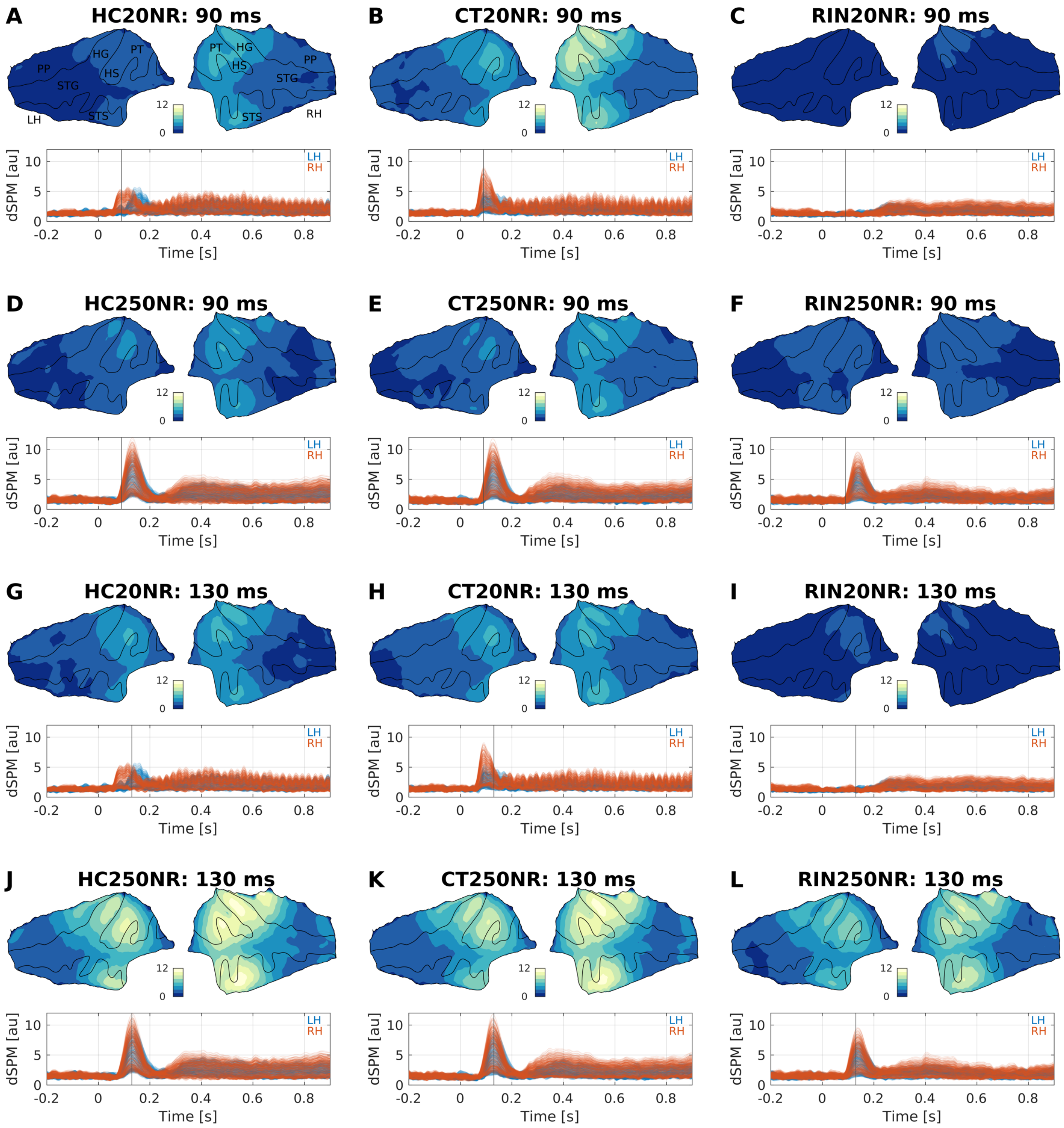

Figure S4. Grand-averaged (n = 10) source time-courses for transition responses. Snapshots of ***Movie S1***. Source activity maps (top) and source time-courses (bottom) are shown for NR (noise-to-regular) transitions at 90 ms (A-F) and 130 ms (G-L) post-transition. Each source time-course line corresponds to a vertex on the supratemporal planes in the left (blue) and right (brown) hemispheres. The color scale is arbitrary thresholded between 0 and 12 (in dSPM arbitrary unit) and discretized to enhance visualization of isocontours. Please refer to the Results section for statistical inference on difference between conditions. Abbreviations: HC, Harmonic complex, CT, click train; RIN, regular interval noise; 20, F0 = 20 Hz; 250, F0 = 250 Hz; NR, noise-to-regular transition; RN, regular-to-noise transition; dSPM, dynamic statistical parametric mapping in arbitrary unit; LH, left hemisphere; RH, right hemisphere; HG,

Heschl's gyrus; HS, Heschl's sulcus; STG, superior temporal gyrus; STS, superior temporal sulcus; PP, planum polare; PT, planum temporale.

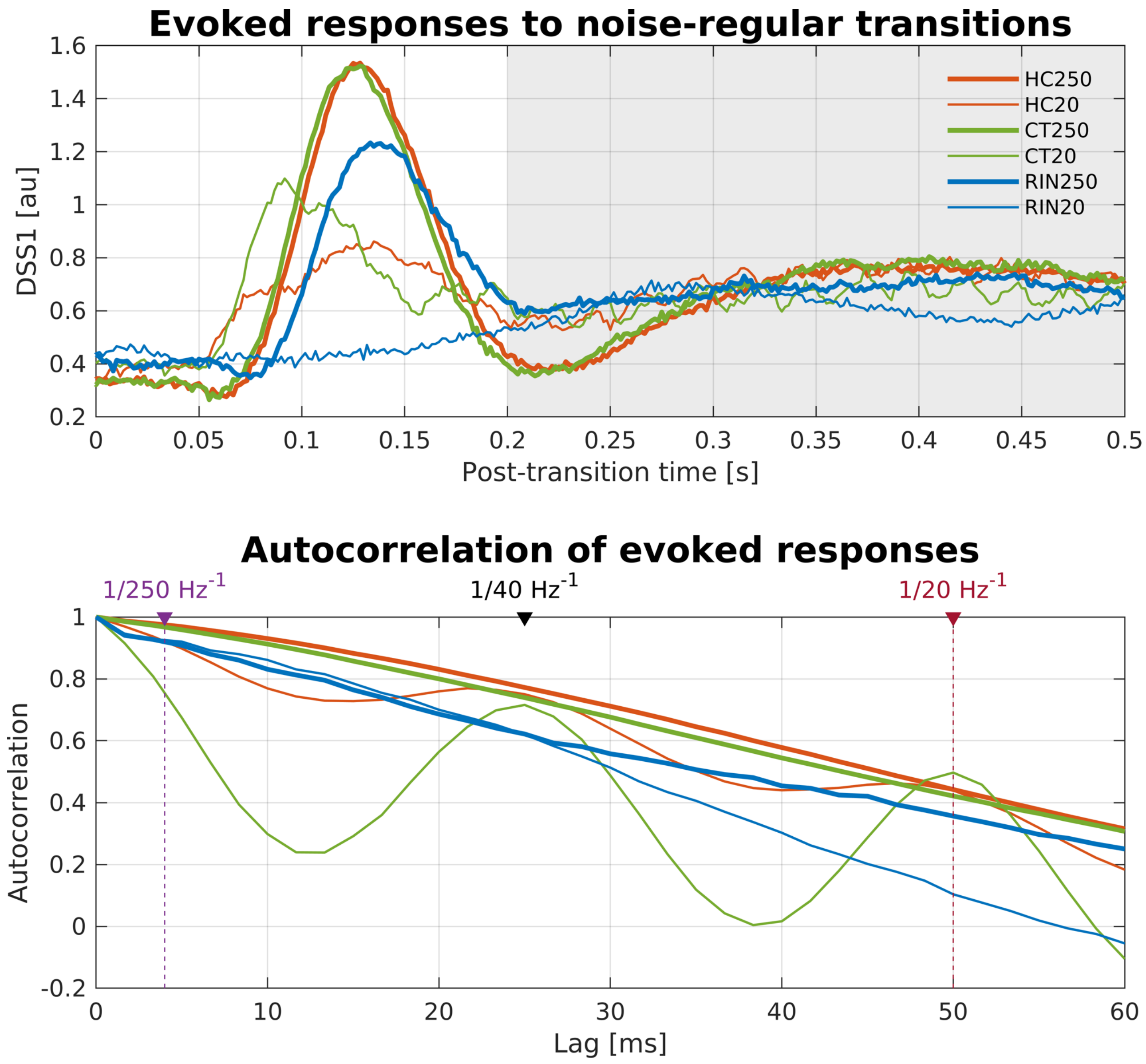

Figure S5. Evoked responses and autocorrelation. Top: evoked responses without low-pass filtering (but with high-pass at 0.5 Hz and notches at harmonics of the 50 Hz power line noise) for noise-to-regular transitions (red, harmonic complex [HC]; green, click-train [CT]; blue, regular-interval noise [RIN]; thick, 250 Hz; thin, 20 Hz). Bottom: autocorrelogram (only positive lags) of the evoked responses computed from 0.2 s to 0.5 s post-transition (shaded in the top panel). The legend is the same as in the top plot.

Transition	***Effect***	***Mean F***	***Time interval [ms]***	***Corrected-p***
NR	Freq	17.85	[-173, -155]	0.0093
NR	Freq	16.79	[-135, -122]	0.0154
NR	Freq	24.08	[58, 83]	0.0040
NR	Freq	52.73	[100, 163]	0.0001
NR	Type	10.30	[-53, -37]	0.0117
NR	Type	29.87	[72, 158]	0.0001
NR	Type	15.23	[363, 470]	0.0002
NR	Type	13.92	[790, 900]	0.0002
NR	Freq x Type	11.38	[58, 92]	0.0029
NR	Freq x Type	11.47	[168, 240]	0.0005
RN	Freq	19.36	[118, 132]	0.0134
RN	Type	14.61	[-113, -88]	0.0036
RN	Type	10.35	[-15, 0]	0.0121
RN	Type	23.51	[52, 83]	0.0010
RN	Type	10.30	[110, 127]	0.0109
RN	Type	18.66	[153, 370]	0.0001
RN	Freq x Type	14.80	[107, 153]	0.0010
RN	Freq x Type	9.34	[342, 353]	0.0152
RN	Freq x Type	12.03	[413, 458]	0.0015
RN	Freq x Type	11.40	[472, 502]	0.0025
RN	Freq x Type	10.05	[550, 563]	0.0118

Table S1. Repeated-measures ANOVA on DSS1 response to transitions. Statistics of significant clusters are given for each cluster in each row. Note that the alpha level was Bonferroni-Holm corrected to control FWER < 0.05. Abbreviations: NR, noise-to-regular; RN, regular-to-noise; Freq, frequency; Type, stimulus type.

Effect	*Mean F*	*Time interval [ms]*	*Corrected-p*
Reg	53.56	[112, 500]	0.0001
Freq	24.63	[35, 72]	0.0018
Freq	43.62	[92, 133]	0.0004
Type	8.75	[130, 133]	0.0403
Reg x Freq	52.67	[85, 143]	0.0001
Freq x Type	12.74	[42, 67]	0.0025
Freq x Type	19.04	[90, 120]	0.0010
Freq x Type	17.09	[142, 237]	0.0001
Reg x Type	10.15	[142, 165]	0.0029
Reg x Freq x Type	17.99	[83, 123]	0.0007
Reg x Freq x Type	12.56	[147, 200]	0.0008

Table S2. Repeated-measures ANOVA on DSS1 response to onsets. Statistics of significant clusters are given for each cluster in each row. Note that the alpha level was Bonferroni-Holm corrected to control FWER < 0.05. Reg, regularity; Freq, frequency; Type, stimulus type.

Cluster index	*Effect*	*Mean F*	*Time interval [ms]*	*Corrected-p*
A	Freq	24.55	[107, 178]	0.0019
B	Type	10.75	[55, 150]	0.0017
C	Type	9.26	[77, 140]	0.0137
D	Type	9.30	[362, 438]	0.0152
E	Freq x Type	7.86	[43, 97]	0.0100
F	Freq x Type	8.99	[70, 102]	0.0184

Table S3*. *Repeated-measures ANOVA on source time-course for NR transitions. Statistics of significant clusters are given for each cluster in each row (cluster indices from ***Figure 6***). Note that the alpha level was Bonferroni-Holm corrected to control FWER < 0.05.

Cluster index	*Effect*	*Mean F*	*Time interval [ms]*	*Corrected-p*
A	Reg	18.60	[103, 147]	0.0067
B	Reg	19.69	[307, 500]	0.0009

Table S4. Repeated-measures ANOVA on source time-course for onsets. Statistics of significant clusters are given for each cluster in each row (cluster indices from ***Figure 7***). Note that the alpha level was Bonferroni-Holm corrected to control FWER < 0.05.

Movie S1. Grand-averaged (n = 10) source time-courses for transition responses. Source activity maps (top) and source time-courses (bottom) are shown for all conditions from 0 to 600 ms post-transition with 10 ms increment. Each source time-course line corresponds to a vertex on the supratemporal planes in the left (blue) and right (brown) hemispheres. The color scale is arbitrary thresholded between 0 and 10 (in dSPM arbitrary unit) and discretized to enhance visualization of isocontours. Please refer to the Results section for statistical inference on difference between conditions. Abbreviations: HC, Harmonic complex, CT, click train; RIN, regular interval noise; 20, F0 = 20 Hz; 250, F0 = 250 Hz; NR, noise-to-regular transition; RN, regular-to-noise transition; dSPM, dynamic statistical parametric mapping in arbitrary unit; LH, left hemisphere; RH, right hemisphere; HG, Heschl's gyrus; HS, Heschl's sulcus; STG, superior temporal gyrus; STS, superior temporal sulcus; PP, planum polare; PT, planum temporale.

Movie S2. Grand-averaged (n = 10) source time-courses for sound-onset-responses. Source activity maps (top) and source time-courses (bottom) are shown for all conditions from 0 to 500 ms post-transition with 10 ms increment. Each source time-course line corresponds to a vertex on the supratemporal planes in the left (blue) and right (brown) hemispheres. The color scale is arbitrary thresholded between 0 and 10 (in dSPM arbitrary unit) and discretized to enhance visualization of isocontours. Please refer to the Results section for statistical inference on difference between conditions. Abbreviations: HC, Harmonic complex, CT, click train; RIN, regular interval noise; 20, F0 = 20 Hz; 250, F0 = 250 Hz; NR, noise-to-regular transition; RN, regular-to-noise transition; dSPM, dynamic statistical parametric mapping in arbitrary unit; LH, left hemisphere; RH, right hemisphere; HG, Heschl's gyrus; HS, Heschl's sulcus; STG, superior temporal gyrus; STS, superior temporal sulcus; PP, planum polare; PT, planum temporale.
