# Supplementary figures and images for: MEG correlates of temporal regularity relevant to pitch perception in human auditory cortex
Source: Neuroimage. 2022 Apr 1;249:118879. doi: 10.1016/j.neuroimage.2022.118879 (PMC8883111; doi:10.1016/j.neuroimage.2022.118879)

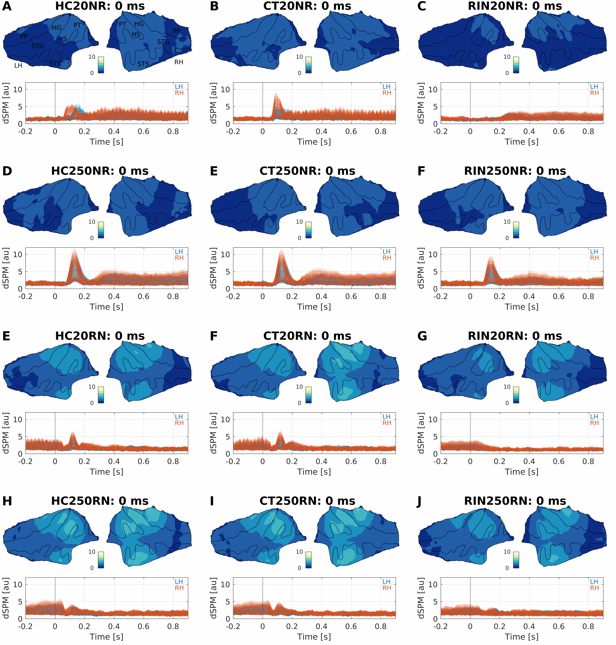

Supplement: Supplementary file 2 — Movie S2. Grand-averaged (n = 10) source time-courses for sound-onset-responses. Source activity maps (top) and source time-courses (bottom) are shown for all conditions from 0 to 500 ms post-transition with 10 ms increment. Each source time-course line corresponds to a vertex on the supratemporal planes in the left (blue) and right (brown) hemispheres. The color scale is arbitrary thresholded between 0 and 10 (in dSPM arbitrary unit) and discretized to enhance visualization of isocontours. Please refer to the Results section for statistical inference on difference between conditions. Abbreviations: HC, Harmonic complex, CT, click train; RIN, regular interval noise; 20, F0 = 20 Hz; 250, F0 = 250 Hz; NR, noise-to-regular transition; RN, regular-to-noise transition; dSPM, dynamic statistical parametric mapping in arbitrary unit; LH, left hemisphere; RH, right hemisphere; HG, Heschl's gyrus; HS, Heschl's sulcus; STG, superior temporal gyrus; STS, superior temporal sulcus; PP, planum polare; PT, planum temporale. [file mmc2.zip › mmc2.gif]

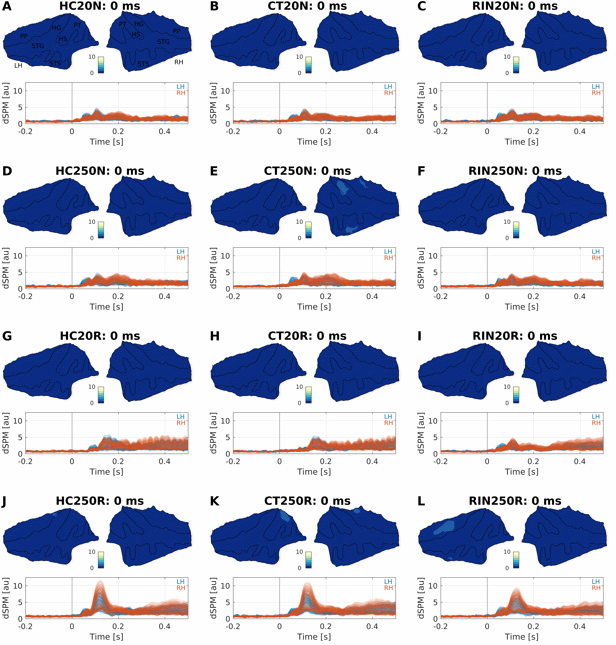

Supplement: Supplementary file 3 [file mmc3.zip › mmc3.gif]
